# Serum unsaturated phosphatidylcholines predict longitudinal basal forebrain degeneration in Alzheimer’s disease

**DOI:** 10.1093/braincomms/fcac318

**Published:** 2022-12-02

**Authors:** Hayley R C Shanks, Kate M Onuska, Dinesh K Barupal, Taylor W Schmitz, for the Alzheimer’s Disease Neuroimaging Initiative, the Alzheimer's Disease Metabolomics Consortium

**Affiliations:** Schulich School of Medicine and Dentistry, University of Western Ontario, London, Ontario, Canada N6A 3K7; Schulich School of Medicine and Dentistry, University of Western Ontario, London, Ontario, Canada N6A 3K7; Department of Environmental Medicine and Public Health, Icahn School of Medicine at Mount Sinai, New York 10029-6574, USA; Schulich School of Medicine and Dentistry, University of Western Ontario, London, Ontario, Canada N6A 3K7; Lawson Health Research Institute, St. Joseph’s Hospital, London, Ontario N6A 4V2, Canada; Robarts Research Institute, Western University, London, Ontario N6A 5B7, Canada; Western Institute for Neuroscience, Western University, London, Ontario N6A 3K7, Canada

**Keywords:** Alzheimer’s disease, cholinergic, nucleus basalis of meynert, phosphatidylcholine, selective vulnerability

## Abstract

Basal forebrain cholinergic neurons are among the first cell types affected by Alzheimer’s disease pathology, but the cause of their early vulnerability is unknown. The lipid phosphatidylcholine is an essential component of the cell membrane, and phosphatidylcholine levels have been shown to be abnormal in the blood and brain of Alzheimer’s disease patients. We hypothesized that disease-related changes in phosphatidylcholine metabolism may disproportionately affect basal forebrain cholinergic neurons due to their extremely large size, plasticity in adulthood and unique reliance on phosphatidylcholine for acetylcholine synthesis. To test this hypothesis, we examined whether serum phosphatidylcholine levels predicted longitudinal basal forebrain degeneration in Alzheimer’s disease. All data were collected by the Alzheimer’s Disease Neuroimaging Initiative. Participants were divided into a normal CSF group (controls; *n* = 77) and an abnormal CSF group (preclinical and clinical Alzheimer’s disease; *n* = 236) based on their CSF ratios of phosphorylated tau and amyloid beta at baseline. Groups were age-matched (*t* = 0.89, *P* > 0.1). Serum lipidomics data collected at baseline were clustered by chemical similarity, and enrichment analyses were used to determine whether serum levels of any lipid clusters differed between the normal and abnormal CSF groups. In a subset of patients with longitudinal structural MRI (normal CSF *n* = 62, abnormal CSF *n* = 161), two timepoints of MRI data were used to calculate grey matter annual percent change for each participant. Multivariate partial least squares analyses tested for relationships between neuroimaging and lipidomics data which are moderated by CSF pathology. Our clustering analyses produced 23 serum lipid clusters. Of these clusters, six were altered in the abnormal CSF group, including a cluster of unsaturated phosphatidylcholines. In the subset of participants with longitudinal structural MRI data, *a priori* nucleus basalis of Meynert partial least squares analyses detected a relationship between unsaturated phosphatidylcholines and degeneration in the nucleus basalis which is moderated by Alzheimer’s disease CSF pathology (*P* = 0.0008). Whole-brain grey matter partial least squares analyses of all 23 lipid clusters revealed that only unsaturated phosphatidylcholines and unsaturated acylcarnitines exhibited an Alzheimer’s disease-dependent relationship with longitudinal degeneration (*P* = 0.0022 and *P* = 0.0018, respectively). Only the unsaturated phosphatidylcholines predicted basal forebrain degeneration in the whole-brain analyses.

Overall, this study provides *in vivo* evidence for a selective relationship between phosphatidylcholine and basal forebrain degeneration in human Alzheimer’s disease, highlighting the importance of phosphatidylcholine to basal forebrain grey matter integrity.

## Teaser

Basal forebrain cholinergic neurons are affected in early stages of Alzheimer’s disease, yet the cause of their vulnerability is unknown. Shanks et al. show that disease-related changes in phosphatidylcholine metabolism disproportionately affect the basal forebrain, indicating a unique link to cholinergic function.

## Graphical Abstract

**Graphical abstract:**
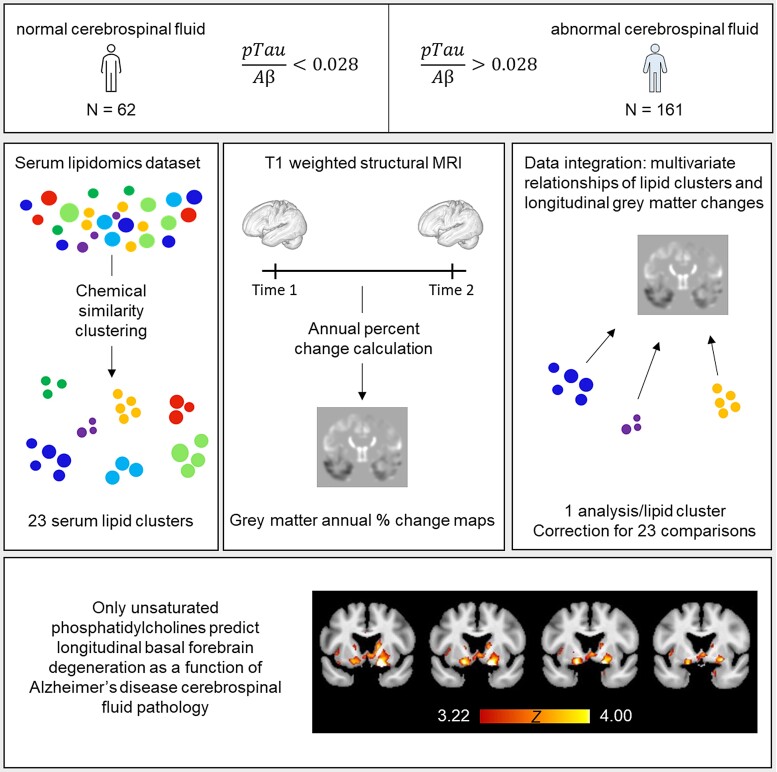

## Introduction

Alzheimer’s disease is characterized by the progressive accumulation of amyloid beta (Aβ) and phosphorylated tau (pTau) in the central nervous system,^1–3^ with certain populations of neurons acquiring these pathologies before others. Among the most vulnerable cell types are the cholinergic neurons of the basal forebrain.^4–8^ Post-mortem histology has long implied that basal forebrain degeneration occurs in individuals with mild cognitive impairment and advanced Alzheimer's disease.^9–12^ More recent longitudinal neuroimaging work^13–16^ and *ex vivo* histology in suspected early Alzheimer's disease patients^5,7,8^ indicates that basal forebrain degeneration begins prior to the onset of clinically detectable cognitive impairment, i.e. the presymptomatic stage of Alzheimer's disease. Cognitively normal older adults at high risk for Alzheimer's disease according to CSF biomarkers of pTau and Aβ exhibit increased basal forebrain degeneration prior to cortical degeneration.^13–16^ Despite the abundant evidence for the selective vulnerability of the cholinergic basal forebrain to Alzheimer's disease degeneration, the cellular characteristics of cholinergic neurons which contribute to their vulnerability remain speculative. One cellular characteristic that is frequently proposed to increase vulnerability to degeneration is the cell’s ‘upkeep cost’, that is, the number of metabolic resources needed for a cell to maintain, repair and build upon its membrane and cytoskeletal structure.^17–20^ Under this hypothetical framework, cholinergic neurons would have higher upkeep costs than other neuronal cell types, and age- or disease-related bottlenecks on the resources needed for upkeep would exacerbate their vulnerability.

Dysregulation in lipid metabolism is one possible bottleneck on normal neuronal upkeep. Like all other neurons in the brain, cholinergic neurons rely on lipid pathways to transport cholesterol and phospholipids for cell membrane repair and construction.^21,22^ One class of lipids in particular, phosphatidylcholines, are an important structural component of the cell membrane, and act as reservoirs for intracellular signalling molecules.^23–25^ An influential theory proposed that cholinergic neurons are selectively vulnerable because they alone additionally use phosphatidylcholines for the biosynthesis of the neurotransmitter acetylcholine.^26^ Recent advances in our understanding of the morphofunctional properties of basal forebrain cholinergic neurons have since strengthened the plausibility of this theory. Cell type-specific labelling studies of basal forebrain cholinergic neurons in mice have revealed the extreme size and extensive branching of these neurons, which in humans are estimated to exceed 100 m in total length for a single neuron.^27,28^ A cell of this size and complexity would have a relatively high demand for phosphatidylcholines to maintain its membrane. Furthermore, acetylcholine signalling pathways from the basal forebrain are critical for the modulation of the neocortex, acting as a core mechanism for enhancing cortical read-in states during the acquisition of novel sensory information and associative learning.^29–34^ Due to their integral role in modulating attention and memory encoding, basal forebrain cholinergic neurons therefore likely remain highly plastic throughout adulthood,^20,35^ continuously remodelling their axonal projections and forming new synapses. Consistent with this idea, the knockdown of axonal phosphatidylcholine synthesis prevents axonal branching in cell culture.^36^ Moreover, in mouse models of Rhett syndrome, disease-related deficits in neuroplasticity are rescued by choline supplementation via increased phosphatidylcholine synthesis.^37^ Considering their large size and sustained plasticity throughout adulthood, age- or disease-related disturbances in phosphatidylcholine metabolism may therefore affect the functioning of basal forebrain cholinergic neurons earlier and more severely than other cell types.

Despite these lines of evidence, the relationship between phosphatidylcholines and basal forebrain cholinergic neuronal integrity remains untested in humans. Non-invasive measures of phosphatidylcholines can be obtained from the blood and have been shown to reliably differentiate older adults at high risk for Alzheimer's disease from age-matched controls.^38^ Furthermore, choline-containing lipids are decreased in both the plasma and brain of Alzheimer's disease patients,^39,40^ indicating that measurements of lipids in the blood may serve as an appropriate proxy for brain lipids. Here we used highly sensitive and specific CSF biomarkers of Alzheimer’s disease pathology^41,42^ to differentiate two age-matched groups of older adults with normal versus abnormal markers of Alzheimer’s pathology.^13,14^ We then integrated longitudinal structural MRI data collected over a 2-year period with a comprehensive panel of serum lipid data collected at MRI baseline. Consistent with our hypotheses, we show that unsaturated phosphatidylcholines predict longitudinal trajectories of degeneration in the basal forebrain and adjacent basolateral nuclei in the abnormal CSF group compared to the normal CSF group. Our results suggest that a disruption of phosphatidylcholine metabolism is associated with the selective vulnerability of cholinergic neurons in Alzheimer’s disease.

## Materials and methods

### ADNI data

Data used in the preparation of this article were obtained from the Alzheimer’s Disease Neuroimaging Initiative (ADNI) database (adni.loni.usc.edu).^43^ The ADNI was launched in 2003 as a public-private partnership, led by Principal Investigator Michael W. Weiner, MD. The primary goal of ADNI has been to test whether serial MRI, PET, other biological markers, and clinical and neuropsychological assessment can be combined to measure the progression of mild cognitive impairment and early Alzheimer’s disease. For up-to-date information, see www.adni-info.org. All ADNI participants gave informed consent according to the Declaration of Helsinki prior to participating in any part of ADNI, and all data collection protocols were approved by the institution where the work was performed. To be included in this study, all participants from ADNI needed to have (i) lipidomics data collected at baseline using the UC Davis lipidomics platform,^44^ (ii) information on body mass index (BMI), and (iii) CSF biomarkers of Aβ and pTau. For analyses relating lipidomics data to neuroimaging measures, participants needed to additionally have longitudinal structural MRI data. Following these exclusion criteria, a total of 313 participants (181 males, 132 females) from ADNI Phase 1 were included in our lipidomics analyses, and 223 participants (127 males, 96 females) were included in our neuroimaging analyses. Participant demographics are outlined in Table 1.

**Table 1:** Participant demographics

	All participants			Longitudinal imaging subset		
	Normal CSF	Abnormal CSF	Statistic	Normal CSF	Abnormal CSF	Statistic
*n* (Total)	77	236		62	161	
CN/MCI/Alzheimer’s disease	77/0/0	29/123/84		62/0/0	25/84/52	
% Male	46.8	61.4	χ^2^ = 5.14*	45.2	61.5	χ^2^ = 4.9*
% APOE4 carriers	10.4	70.3	χ^2^ = 84.52***	12.9	71.4	χ^2^ = 62.0***
Age	75.30 (5.50)	74.60 (7.32)	*t* = 0.89	75.1 (5.3)	74.5 (7.1)	*t* = 0.6
Body mass index	27.20 (4.23)	25.37 (3.67)	*t* = 3.66***	27.3 (4.5)	25.6 (3.7)	*t* = 2.78**
ADNI composite memory	0.10 (0.52)	−0.36 (0.73)	*t* = 17.71***	1.0 (0.5)	−0.3 (0.8)	*t* = 14.42***
ADNI composite executive function	0.72 (0.70)	−0.41 (0.92)	*t* = 11.39***	0.69 (0.68)	−0.33 (0.95)	*t* = 8.95***
Education	15.66 (2.68	15.49 (3.09)	*t* = 0.44	15.6 (2.8)	15.6 (3.0)	*t* = 0.19

Demographic and cognitive information on ADNI participants by CSF group. All values are presented as mean (standard deviation) unless otherwise specified. Independent samples *t*-tests (*t*) and χ^2^ tests were used to assess CSF group differences for the full sample and sub-sample of participants. ADNI = Alzheimer’s Disease Neuroimaging Initiative; APOE = apolipoprotein E; CN = cognitively normal; MCI = mild cognitive impairment. *P < 0.05; **P < 0.01; ***P < 0.0001.

### CSF biomarker grouping strategy

CSF Aβ and pTau were collected for ADNI and quantified as described previously.^42,45^ Participants were split into groups based on their ratio of CSF pTau/Aβ at baseline. The ratio of pTau/Aβ has been shown to be a sensitive and specific predictor of Alzheimer's disease pathology and tracks with Aβ PET imaging.^41,42^ Participants with ratios of pTau/Aβ below 0.028 are within the normal range^41^ and were therefore considered the normal CSF group, while participants with ratios above 0.028 were considered the abnormal CSF group.

Clinical diagnosis in ADNI is determined using standardized criteria involving comprehensive neuropsychological testing and clinical examination by a neurologist.^46^ Participants with normal CSF but clinically detectable cognitive impairment were excluded from this study because their cognitive dysfunction likely stems from another form of dementia, such as vascular dementia.^47^

### Serum lipidomics data

Serum lipidomics data were collected through untargeted ultra-high-performance liquid chromatography quadrupole time-of-flight mass spectrometry. For information on serum lipidomics data collection and processing in ADNI, see.^44^ Of the 521 measured lipids from the complete lipidomics data set available on ADNI, we excluded all unannotated lipids (lipids that lacked names and chemical identifiers) and one duplicate lipid. The final data set used in this study consisted of 348 annotated lipids. Participants’ serum levels of each lipid were standardized via *z*-score prior to all statistical analyses to allow for comparisons across lipid species.

### Lipid clustering by chemical similarity

ADNI lipids were clustered based on structural similarity and chemical ontology using chemical similarity enrichment analysis (ChemRICH) with chemical classes as the set definition.^48^ ChemRICH first maps each lipid to its most specific Medical Subject Heading term to reflect chemical ontology, then modifies clusters by comparing binary representations of the structure of compounds, called substructure fingerprints.^49^ The Tanimoto coefficient is used to create a pairwise similarity matrix for all lipids based on their substructure fingerprint, which then undergoes hierarchical clustering. Compounds with over 85% similarity are grouped into the same cluster.^48^ After clustering has been initially performed, Dynamic Tree Cutting^50^ determines if any sub-clusters exist within these generated clusters. Because ChemRICH only relies on the chemical structure of the lipids in the data set, it will produce the same lipid clusters in different sets of participants, provided both samples share a common lipidomics platform.

After the formation of lipid clusters, we used the ChemRICH software to perform cluster set enrichment analysis.^48^ First, we compared serum levels of each lipid in the normal and abnormal CSF groups using independent samples Wilcoxon tests. The Wilcoxon *P* values (two-tailed) were aggregated for each lipid cluster to form a distribution. ChemRICH then tests the null hypothesis that this cluster-level distribution of *P* values are obtained from a reference uniform *P* distribution using the Kolmogorov–Smirnov (KS) test.^48^ If the KS test rejects the null hypothesis for a lipid cluster, this would indicate that a cluster of lipids is significantly altered in the abnormal CSF group compared with the normal CSF group. For visualization of the direction of change in lipid levels between the normal and abnormal CSF groups, we additionally determined whether lipids were upregulated or downregulated in the abnormal CSF group. To do so, we divided the median serum level of each lipid in the abnormal CSF group by the median level in the normal CSF group to produce a fold change estimate.

### Longitudinal structural MRI

A subset of participants with lipidomics, CSF and longitudinal structural MRI data (*n* = 223) were included in all further analyses. Longitudinal structural MRI data were collected at baseline and ∼ 6-month follow-ups.^51^ Preprocessing and annual percent change calculations were performed as part of a previous study.^14^ First, all structural MRI images were co-registered to tissue probability maps which have been enhanced to improve the characterization of the subcortical grey matter.^52^ Longitudinal structural MRI data were analyzed using the SPM12 serial longitudinal pipeline.^53^ For each participant a within-subject symmetric mid-point average image was created using two timepoints (∼ 2 years apart) of their longitudinal T_1_-weighted imaging data. The interscan interval in years was used to regularize the deformations during the creation of the within-subject mid-point averages. A Jacobian determinant which represents how much each voxel has contracted or expanded compared with the mid-point average was written out for each time point. The within-subject average images were segmented and bias-corrected using SPM12’s segment with the enhanced tissue probability maps.^52^ The grey matter segment of each participant’s mid-point average image was multiplied by the Jacobian determinant for each time point to produce longitudinally modulated grey matter segments which provide an estimate of within-subject change over time.^53^ Next, the rigidly transformed grey matter and white matter segments for each individual were used to create a custom, age-appropriate template for the ADNI population.^54^ After the creation of the ADNI template, a deformation field was estimated between the template and the within-subject mid-point average image for each participant. Each participant's deformation field was then applied to each modulated grey matter segment to warp them into the template space for comparison across individuals. Finally, the voxel-wise annual percent change of grey matter volume was calculated using the warped longitudinally modulated grey matter segments for each individual:

(1) $$\begin{array}{rl} \mathrm{annual}\;\mathrm{percent}\;\mathrm{change} & =\left(\frac{\mathrm{change}\;\mathrm{baseline}\;(\mathrm{ml})}{\mathrm{value}\;\mathrm{baseline}\;(\mathrm{ml})}\right)\\ & \;\times \left(\frac{365}{\mathrm{interscan}\;\mathrm{interval}\;(\mathrm{days})}\right)\\ & \;\times 100 \end{array}$$

This formula was applied to produce annual percent change maps which show the annual percent change at each voxel in the whole-brain grey matter for each individual in the ADNI template space. Using annual percent change as our measure of longitudinal degeneration controls for any differences in the interval between MRI scans across participants.

### Region of interest definition

**Figure 1:**
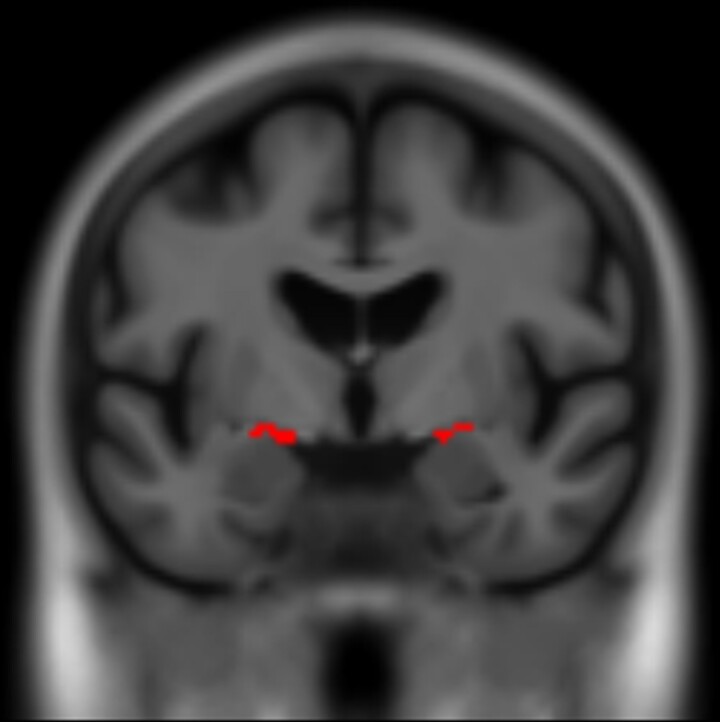
**NbM region of interest.** The NbM (Ch4 and Ch4p) region of interest is shown overlaid on a coronal slice in the ADNI population template space. The underlay is the produced from averaging all subjects’ T_1_-weighted MRI scan in template space (see methods).

Based on our prior work,^13,14^ we defined the nucleus basalis of Meynert (NbM) as an *a priori* region of interest. The NbM consists of Ch4 and Ch4p according to Mesulam’s nomenclature.^55^ The NbM is composed of over 90% cholinergic neurons^56,57^ which are known to be especially vulnerable to Alzheimer's disease pathology.^7,8,14–16,58,59^ A stereotaxic probabilistic map created through post-mortem histology was used to isolate voxels in the NbM.^60^ The NbM probabilistic map from the left and right hemispheres was warped from the Montreal Neurological Institute space into the ADNI population template space. Only voxels that overlapped in 50% of donors were used in the final NbM mask (Fig. 1).

In addition, the whole-brain grey matter was included in our analyses to test the anatomical specificity of any relationships seen in the NbM region of interest analyses. To define our whole-brain grey matter region, we binarized the ADNI population template grey matter segment to include only voxels with at least a 30% probability of belonging to the grey matter.

### Statistical analyses

#### *A priori* basal forebrain sub-region of interest partial least squares analyses

To test our *a priori* hypothesis that phosphatidylcholines predict basal forebrain degeneration, we examined multivariate relationships between phosphatidylcholines and NbM grey matter degeneration in probable Alzheimer's disease using multivariate partial least squares (PLS) correlation analyses.^61,62^ PLS is a form of pattern analysis that searches for orthogonal latent variables which represent a significant covariance pattern between two input datasets that differs as a function of a grouping variable. Here, we used PLS analyses to assess relationships between lipids and grey matter annual percent change within the NbM region of interest with CSF pathology as the grouping factor. Age and BMI were included as input variables. For a detailed description of the methodological implementation of PLS analysis, see.^61^

The significance of distinct latent variables in PLS models was assessed using 5000 permutation tests. PLS analyses are not directional^61^ and therefore all statistics were performed two-tailed. An alpha of *P* < 0.05 was used in all analyses. Bootstrapping (5000 iterations) was used to derive 95% confidence intervals for each input variable to PLS analyses. These confidence intervals demonstrate how reliably each variable contributed to latent variable(s) identified in each PLS analysis.^61^

#### Whole-brain partial least squares analysis

Next, we assessed the biochemical and anatomical specificity of any relationships detected between phosphatidylcholines and basal forebrain grey matter integrity. To assess biochemical specificity, we conducted one PLS analysis per ChemRICH lipid cluster. To assess anatomical specificity, we expanded the search space to the whole-brain grey matter. With the exception of the search region, all input specifications for these PLS analyses were the same as the NbM analyses.

### Data availability

All raw data used in this study are freely available from https://ida.loni.usc.edu. The ChemRICH analysis was performed in R. The code to perform ChemRICH can be found online (https://github.com/barupal/ChemRICH). Longitudinal structural MRI preprocessing was performed in MATLAB using SPM12 (https://www.fil.ion.ucl.ac.uk/spm/software/download/). PLS analyses were performed in MATLAB version 2019b, using PLS software which is freely available (https://www.rotman-baycrest.on.ca).

## Results

### Group characteristics

ADNI participants included in this study had CSF pTau/Aβ and serum lipidomics data which were collected at baseline. For our analyses using structural MRI data, (*n* = 223) participants additionally had to have two structural MRI scans which were collected ∼2 years apart. Only participants with complete data across all three modalities were included. For all analyses, participants were broken into a normal CSF group (CSF pTau/Aβ < 0.028, full sample *n* = 77, longitudinal MRI subset *n* = 62) and an abnormal CSF group (CSF pTau/Aβ > 0.028, full sample *n* = 236, longitudinal MRI subset *n* = 161). The normal CSF group consists of cognitively normal individuals without CSF-based evidence of Alzheimer’s disease pathology, while the abnormal CSF group consists of participants with various clinical cognitive diagnoses (cognitively normal, mild cognitive impairment, and Alzheimer’s disease) and CSF biomarkers consistent with Alzheimer’s disease. Table 1 summarizes participant demographics and clinical characteristics in the full sample and MRI subset. Importantly, in both the full sample and MRI subset, CSF groups were matched on age and number of years of education (*P* > 0.05 for all). In the full sample of participants and the MRI subset, the frequency of carriers of the apolipoprotein E (*APOE*) ε4 allele was significantly higher in the abnormal CSF group (*P* < 0.001 in both the full sample and MRI subset). Consistent with expectations, the abnormal CSF group in both participant samples had worse memory and executive functioning performance than the normal CSF group (*P* < 0.001 for all). Test statistics and *P* values for between-group comparisons of demographic and clinical variables are presented in Table 1.

### Chemical similarity enrichment analysis

ChemRICH^48^ was applied to the ADNI serum lipidomics dataset to form lipid clusters based on chemical ontology and structural similarity. The ChemRICH analysis produced 23 lipid clusters. Of these, ChemRICH delineated two clusters of phosphatidylcholines: saturated phosphatidylcholines and unsaturated phosphatidylcholines. ChemRICH clusters ranged in size from three to 76 lipids. Two lipids were not assigned to any lipid cluster after ChemRICH and were excluded from further analyses.

After the formation of lipid clusters, ChemRICH was used to determine whether the serum levels of any of the lipid clusters differed in the normal and abnormal CSF groups. To do this, we compiled *P* values derived from univariate Wilcoxon tests for each lipid in a given cluster. The distribution of *P* values from the cluster is then compared to a theoretical uniform *P* distribution.^48^ If the cluster *P* distribution differs significantly from the uniform *P* distribution, this suggests that the serum levels of lipids in that cluster differ between the normal and abnormal CSF groups. This methodology is ideal for lipidomic analyses, as it provides information on how individual lipid species are dysregulated by pTau/Aβ while also providing statistics at the cluster level. This allows patterns of effects common across chemically similar lipid species to be detected.

**Figure 2:**
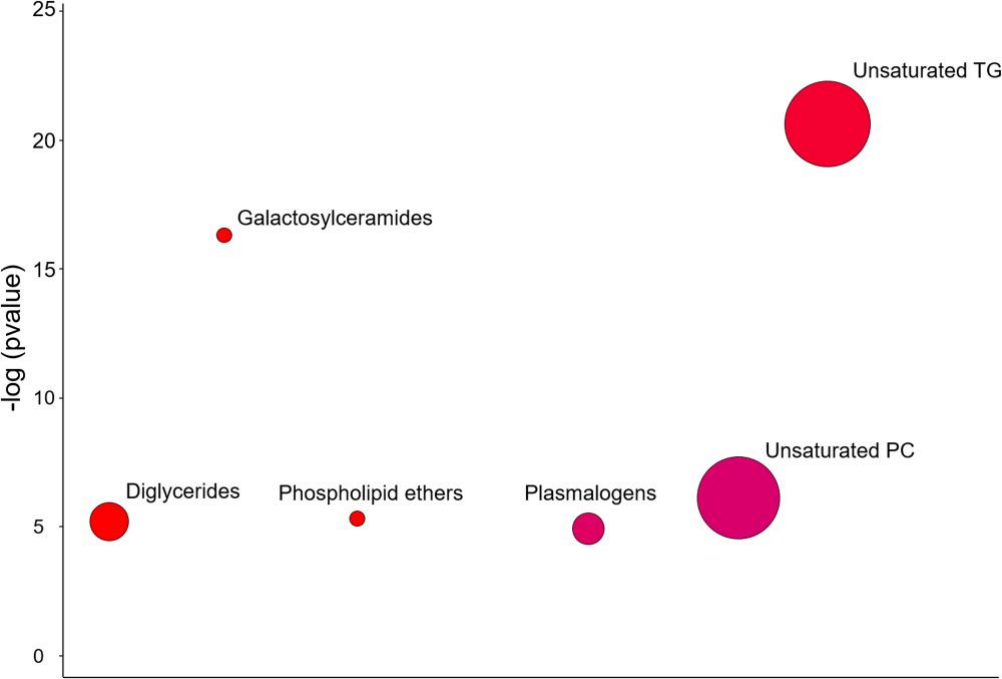
**Chemically similar lipid clusters dysregulated by pTau/Aβ pathology.** ChemRICH revealed alterations in serum lipid clusters in the abnormal CSF group (*n* = 236) when compared with the normal CSF group (*n* = 77). Only lipid clusters with ­*p* < 0.05 after FDR correction for 23 comparisons across lipid clusters are shown. Circumference of the dots representing lipid clusters are proportional to the number of lipids in that cluster. The colour of the dot represents whether the lipids within each cluster are increased overall (red), decreased overall (blue) or mixed (magenta). Dots are organized alphabetically along the *x*-axis.

Outputs from the ChemRICH analysis are summarized in Table 2, including cluster sizes, *P* values, and directions of the effects detected. After correction for multiple comparisons across 23 lipid clusters, six serum lipid clusters were significantly altered by CSF pTau/Aβ: unsaturated triglycerides, galactosylceramides, phospholipid ethers, unsaturated phosphatidylcholines, diglycerides, and plasmalogens (Fig. 2). In three of these lipid clusters (galactosylceramides, phospholipid ethers, and diglycerides), all lipids which were significantly altered in the abnormal CSF group were increased (Fig. 2, red clusters). In the plasmalogen, unsaturated triglyceride, and unsaturated phosphatidylcholine clusters, some lipid species were increased while others decreased (Fig. 2, magenta clusters). For example, four unsaturated phosphatidylcholines were increased while six were decreased in the abnormal CSF group (Table 2). This suggests that pTau/Aβ pathology causes dysregulation of multiple serum lipid clusters, including phosphatidylcholines, and that pathology may have bi-directional effects on the metabolism of individual lipid species even when they are structurally similar.

**Table 2:** Chemical similarity enrichment analysis results

Cluster name	Cluster size	Cluster *P*-value	FDR *P*-value	Key compound	Altered lipids	Increased	Decreased
Unsaturated triglycerides	76	1.100E-09	2.500E-08	TG(18:2_18:2_22:5)	19	17	2
Galactosylceramides	5	8.200E-08	9.400E-07	GlcCer(d41:1)	5	5	0
Phospholipid ethers	3	0.0014	0.010	PC(p-40:4)	1	1	0
Unsaturated phosphatidylcholines	70	0.0021	0.012	PC(p-18:0_20:3)	10	4	6
Diglycerides	13	0.0055	0.025	DG(16:0_18:1)	3	3	0
Plasmalogens	9	0.0071	0.027	PE(p-40:7)	2	1	1
Saturated fatty acids	13	0.017	0.056	FA(26:0)	1	1	0
Phosphatidylinositols	11	0.02	0.057	PI(18:0_22:6)	2	2	0
Unsaturated fatty acids	16	0.025	0.063	FA(22:1)	3	1	2
Saturated triglycerides	8	0.092	0.210	TG(14:0_16:0_16:0)	0	0	0
Saturated acylcarnitines	4	0.1	0.220	AC(18:0)	0	0	0
Unsaturated acylcarnitines	5	0.4	0.730	AC(18:2)	1	1	0
Cholesterol esters	8	0.41	0.730	CE(18:2)	1	0	1
Unsaturated ceramides	18	0.62	1	Gal-Gal-Cer(d18:1/16:0)	1	1	0
Ethanolamines	7	0.72	1	LPE(20:4)	1	1	0
Lysophospholipids	3	1	1	LPC (p-16:0)	0	0	0
Saturated sphingomyelins	6	1	1	SM(d42:0)	0	0	0
Phosphatidylethanolamines	14	1	1	PE(18:0_22:6)	0	0	0
Saturated ceramides	3	1	1	Cer(d42:0)	0	0	0
Saturated lysophosphatidylcholines	7	1	1	LPC(14:0)	0	0	0
Saturated phosphatidylcholines	7	1	1	PC(16:0_18:0)	0	0	0
Unsaturated sphingomyelins	26	1	1	SM(d18:1_16:0)	0	0	0
Unsaturated lysophosphatidylcholines	14	1	1	LPC(20:3)	0	0	0

Results from chemical similarity enrichment (ChemRICH) analysis comparing serum lipid clusters in the normal (*n* = 77) and abnormal (*n* = 236) CSF groups. Wilcoxon rank sum tests were conducted on each lipid species in the normal and abnormal CSF groups. *P* values from these comparisons were compiled into a distribution for each lipid cluster, and compared to the uniform *p* distribution using a Kolmogorov–Smirnov test. Significant cluster *p* values indicate that a lipid cluster is dysregulated in the abnormal CSF group. The key compounds from each cluster which are most significantly altered in the abnormal CSF group compared to the normal CSF group are named by their lipid class abbreviation in capital letters. The number of carbons and double bonds on any side chains are included in parentheses as (carbons:bonds). Underscores indicate separate chains, and alphabetical characters in parentheses indicate bond types. Lipids are presented as being increased or decreased in the abnormal CSF group relative to the normal CSF group. AC = acylcarnitine, CE = cholesterol ester, Cer = ceramide, d = dihydroxyl, DG = diglyceride, FA = fatty acid, FDR = false discovery rate, Gal = galactosyl, GlcCer = glucosyl ceramide, LPC = lysophosphatidylcholines, LPE = lysophosphatidylethanolamine, o = alkyl bond, p = alkenyl bond, PC = phosphatidylcholine, PE = phosphatidylethanolamine, SM = sphingomyelin, TG = triglyceride.

### Serum phosphatidylcholines are associated with NbM degeneration in the presence of Alzheimer’s disease pathology

After determining that serum unsaturated phosphatidylcholines are significantly altered in individuals with abnormal CSF pTau/Aβ, we used PLS correlation analyses^61,62^ to test our *a priori* hypothesis that phosphatidylcholines relate to the grey matter integrity of the basal forebrain in Alzheimer’s disease. In our analysis, PLS tested whether there are unique components of covariance (called latent variables) shared between unsaturated phosphatidylcholines and degeneration of the NbM. In prior post-mortem histological^7,8,12,59^ and *in vivo* neuroimaging^14,15,63–66^ research, the NbM has been consistently identified as the most vulnerable nucleus of the basal forebrain to neurodegeneration. Previous research has detected relationships between individual lipid species and grey matter degeneration using univariate statistical methods.^67,68^ However, such as genes and proteins, lipid species are highly interrelated and form complex regulatory networks.^69,70^ For this reason, we used PLS to examine the multivariate relationships of biochemically defined ChemRICH lipid clusters with longitudinal grey matter degeneration. PLS analyses used permutation testing^61^ to test if the pattern of covariance between lipids and brain degeneration differs by a grouping factor. When this is true, significant latent variable(s) will be produced.

PLS analyses can have three possible outcomes: no significant latent variables, one significant latent variable, or multiple significant latent variables. If there is no significant latent variable, then either no multivariate relationship is detected in either group or the multivariate relationships are symmetrical in both groups (null hypothesis is supported). Alternatively, if one or more significant latent variables is detected, then a significant multivariate relationship exists between baseline lipids and longitudinal grey matter degeneration which differs as a function of CSF group. Significant latent variables are then expressed as a salience map, which quantifies the multivariate relationship between baseline lipids and longitudinal grey matter degeneration at each voxel.^61^ These salience maps are similar to *z*-scores, where values farther from zero represent a stronger relationship between baseline lipids and grey matter degeneration in a given voxel.

For our primary analyses, participants were split by CSF group to determine whether there is a relationship between serum lipid levels and longitudinal grey matter degeneration which is moderated by CSF-confirmed Alzheimer's disease pathology. Due to the impact of age and BMI on lipid metabolism^71^ and neurodegeneration,^72–75^ we additionally included these variables in the PLS analyses to examine their contribution to the covariance pattern.

**Figure 3:**
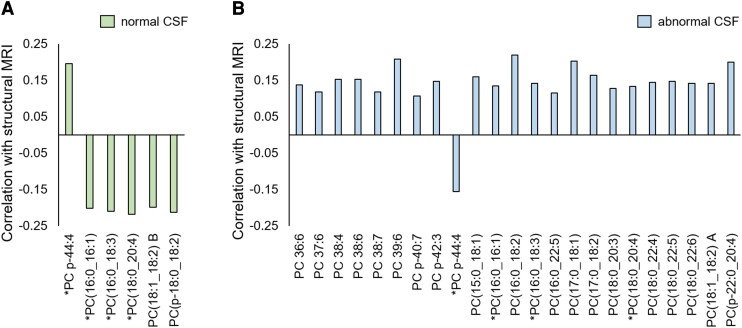
**Correlations between unsaturated phosphatidylcholines and NbM annual percent change derived from PLS analyses.** PLS analyses examined relationships between serum unsaturated phosphatidylcholines and longitudinal NbM grey matter degeneration in the normal (*n* = 62) and abnormal (*n* = 161) CSF groups. A significant latent variable was detected (*P* = 0.0008 on 5000 permutations), indicating a significant relationship between unsaturated phosphatidylcholines and NbM annual percent change which is moderated by CSF pathology. Correlation analyses were used to assess how strongly each individual unsaturated phosphatidylcholine related to the PLS latent variable in the normal and abnormal CSF groups. Five-thousand iterations of bootstrapping were performed on each correlation to assess reliability. Lipids producing a reliable correlation in the normal CSF group are shown in (**A**), and lipids producing a reliable correlation with the PLS latent variable in the abnormal CSF group are shown in (**B**). Lipids which produced a reliable correlation with the latent variable in both the normal and abnormal CSF groups are indicated with a *. Lipids are named according to their headgroup [phosphatidylcholine (PC)] and total carbons and double bonds (carbons:double bonds) on their acyl chains. Acyl chain information is indicated for lipids when available. Isomer compounds are distinguished with an ‘A’ or ‘B’ following their name. p = alkenyl bond.

Although the ChemRICH clustering analysis delineated two clusters of phosphatidylcholines (saturated and unsaturated), we focused our *a priori* NbM PLS analyses on the unsaturated phosphatidylcholines, as these lipids were the only phosphatidylcholine cluster which was significantly dysregulated in the abnormal CSF group (Table 2). Consistent with our hypothesis for a molecular basis of phosphatidylcholine metabolism in cholinergic basal forebrain integrity, we found that the PLS analysis produced a single significant latent variable. Specifically, baseline levels of serum unsaturated phosphatidylcholines predicted longitudinal degeneration within the NbM (*P* = 0.0008 on 5000 permutation tests). Correlations between all lipids in the unsaturated phosphatidylcholine cluster and their bootstrapped confidence intervals are shown in Supplementary Table 1. We examined whether each unsaturated phosphatidylcholine in the cluster correlated reliably with the latent variable (95% bootstrapped confidence intervals for the correlation does not cross zero) in one or both CSF groups. Of the reliable correlations (Fig. 3), 76% occurred in abnormal CSF only, 8% occurred in normal CSF only, and 16% occurred in both groups. The predominance of significant relationships in the abnormal CSF group suggests that covariance between unsaturated phosphatidylcholines and NbM degeneration is driven by the presence of Alzheimer's disease CSF proteopathies (pTau/Aβ). In terms of the biochemical structure of the significant unsaturated phosphatidylcholines, 80% were diacyl phosphatidylcholines, as opposed to alkyl or alkenyl phosphatidylcholines. Moreover, 88% of reliable phosphatidylcholines contained polyunsaturated fatty acids, including arachidonic acid, docosahexaenoic acid and docosapentaenoic acid (Fig. 3). Overall, this result highlights the importance of polyunsaturated fatty acid-containing diacyl phosphatidylcholines to CSF-confirmed Alzheimer's disease degeneration of the NbM. Thus, in line with our hypothesis, the CSF pTau/Aβ grouping factor differentiated multivariate relationships of phosphatidylcholines with longitudinal NbM degeneration.

### Serum phosphatidylcholines are associated with degeneration in the basal forebrain cholinergic projection system

The basal forebrain sends dense cholinergic projections to multiple brain regions. Recent *in vivo* PET studies with the ^18^F-Fluoroethoxybenzovesamicol ([^18^F]-FEOBV) radiotracer, which binds to the vesicular acetylcholine transporter, has revealed that the densest cholinergic innervations in the human brain include the striatum, lateral and medial temporal cortices, cingulate cortex, insula, and amygdala.^76,77^ Our recent work suggests that longitudinal degeneration within the NbM covaries with cortico-amygdalar topographies of both structural degeneration and cholinergic denervation, and, that this covariation reflects the organization of the basal forebrain cholinergic projections.^47^ We therefore conducted PLS analyses examining the multivariate relationship between baseline phosphatidylcholines and longitudinal grey matter degeneration throughout the entire brain, with the prediction that this relationship would be most robustly expressed by targets of the basal forebrain cholinergic projections.

**Figure 4:**
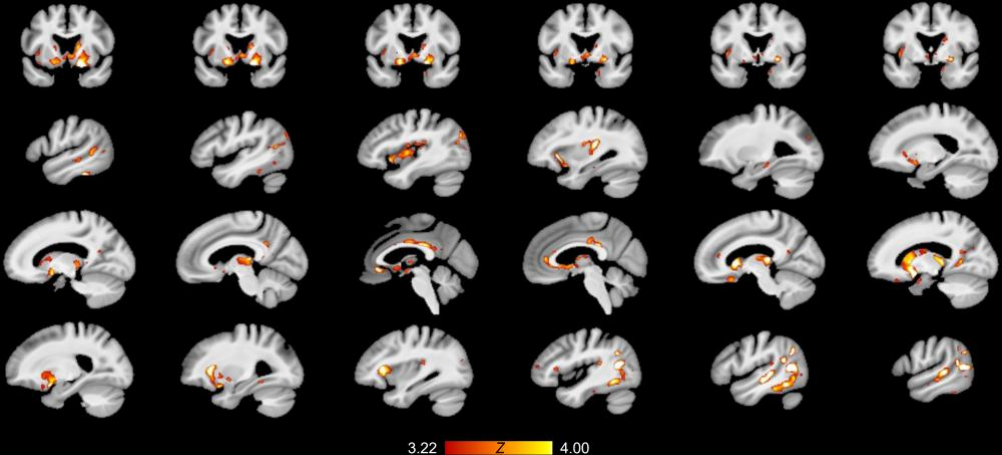
**Spatial patterns of grey matter degeneration predicted by baseline levels of serum unsaturated phosphatidylcholines.** Whole-brain grey matter PLS analyses revealed that serum levels of unsaturated phosphatidylcholines at baseline predict longitudinal degeneration as a function of Alzheimer’s disease CSF pathology (latent variable *P* = 0.0022; normal CSF group *n* = 62, abnormal CSF group *n* = 161). The salience map showing the expression of this latent variable is overlaid on the structural images. Grey matter voxels which have significant saliences after FDR correction (*P* < 0.05; shown in warm tones) are voxels which exhibit a significant, reliable relationship with unsaturated phosphatidylcholines as a function of pTau/Aβ pathology. Unsaturated phosphatidylcholines predict Alzheimer’s disease-related degeneration of the basal forebrain, striatum, anterior and posterior cingulate and temporal cortex. Expression of the salience map within the basal forebrain nuclei is most evident in the coronal slices (top row), where the saliences visible in this view are predominantly in the basal forebrain.

The whole-brain grey matter PLS analysis revealed one significant latent variable for the unsaturated phosphatidylcholines (*P* = 0.0022 on 5000 permutations). Figure 4 shows the salience map resulting from the unsaturated phosphatidylcholine after correction for multiple comparisons throughout voxels in the grey matter using the false discovery rate (FDR) correction.^78^ This salience map depicts the expression of the PLS latent variable relating unsaturated phosphatidylcholines to grey matter degeneration throughout the brain. Grey matter voxels that have significant saliences after FDR correction are voxels that exhibit a significant, reliable relationship with unsaturated phosphatidylcholines as a function of pTau/Aβ pathology. The salience map demonstrates that in addition to the basal forebrain, unsaturated phosphatidylcholines predict degeneration as a function of pathological pTau/Aβ in cortical regions that closely match the projections of the cholinergic system.^76,77^ Consistent with the [^18^F]-FEOBV PET work, these regions include the basal forebrain, caudate nucleus, thalamus, hippocampus, insula, temporal cortex, and anterior and posterior cingulate. Overall, in line with our NbM analysis, our whole-brain PLS analysis suggests that unsaturated phosphatidylcholines are important for the integrity of the basal forebrain and cortical and subcortical areas targeted by its projections.

### Phosphatidylcholines predict Alzheimer’s disease degeneration with high biochemical specificity

We next aimed to assess the biochemical specificity of the observed relationship between baseline serum unsaturated phosphatidylcholine levels and longitudinal grey matter degeneration. To do so, we conducted PLS analyses on the remaining 22 ChemRICH lipid clusters. Each of the 22 PLS analyses examined the multivariate relationship between baseline lipids (for a given cluster) and longitudinal degeneration throughout the whole-brain grey matter tissue compartment.

**Figure 5:**
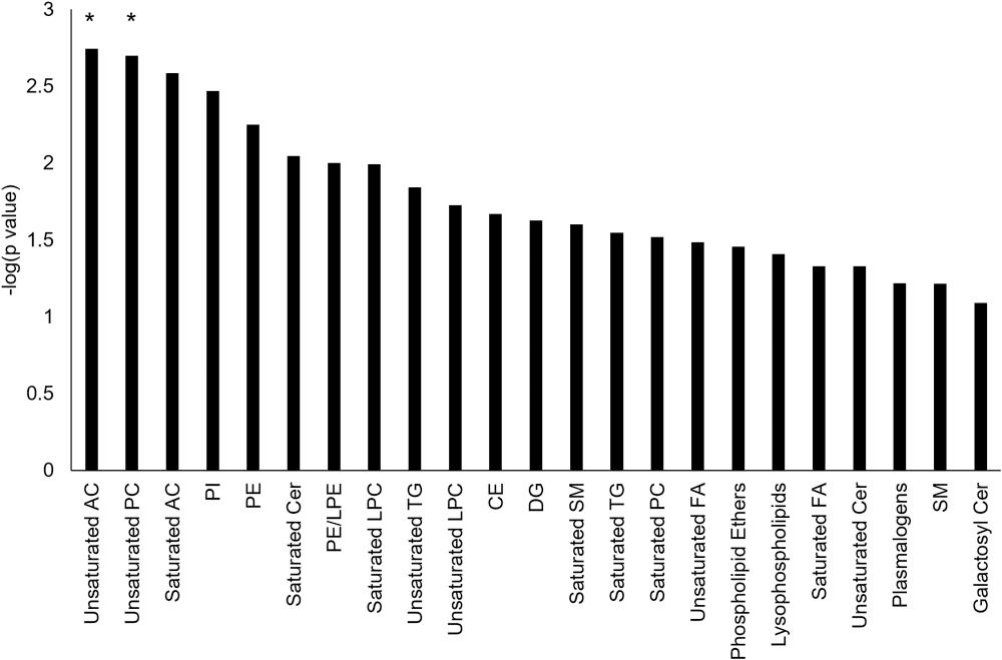
**Summary of whole-brain grey matter PLS analyses by chemRICH lipid cluster.** PLS analyses tested whether each lipid cluster predicted longitudinal grey matter degeneration as a function of CSF group (normal CSF *n* = 62, abnormal CSF *n* = 161). Lipid clusters with a significant latent variable after using the Bonferroni correction to adjust the significance threshold for 23 comparisons (*P* < 0.05/23) are marked with a *. The number of lipids in each lipid cluster is listed in Table 2. AC = acylcarnitine; CE = cholesterol ester; Cer = ceramide; DG = diglyceride; FA = fatty acid; LPC = lysophosphatidylcholine; LPE = lysophosphatidylethanolamine; PC = phosphatidylcholine; PE = phosphatidylethanolamine; PI = phosphatidylinositol SM = sphingomyelin; TG = triglyceride.

**Figure 6:**
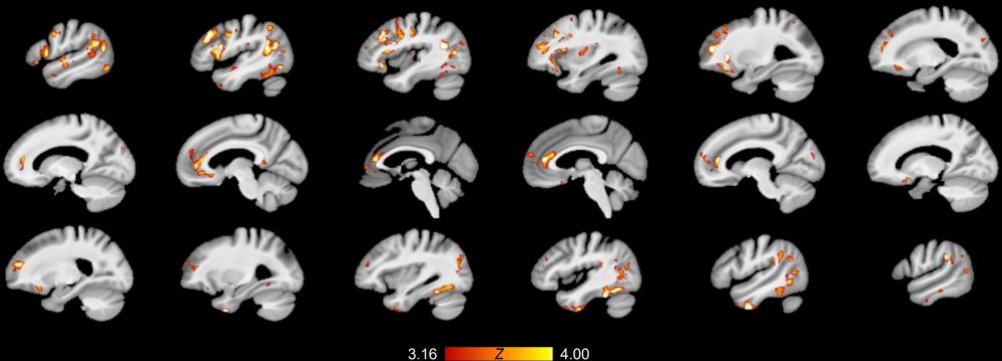
**Spatial patterns of degeneration predicted by baseline levels of serum unsaturated acylcarnitines.** Serum levels of unsaturated acylcarnitines predict different trajectories of longitudinal degeneration (latent variable *P* = 0.0018) in the normal (*n* = 62) and abnormal (*n* = 161) CSF groups through whole-brain PLS analyses. Regions expressing a significant relationship with unsaturated acylcarnitines are shown in warm tones. These regions are primarily cortical, including the inferior temporal cortex, anterior cingulate and prefrontal cortex. Only voxels with a *P* < 0.05 after FDR correction for all voxels in the grey matter are shown.

We used the Bonferroni correction to adjust the significance threshold for the total number of whole-brain CSF PLS analyses conducted. Unsaturated phosphatidylcholines (*P* = 0.0022 on 5000 permutations) and unsaturated acylcarnitines (*P* = 0.0018 on 5000 permutations) were the only lipid clusters that produced a significant latent variable (Fig. 5). This demonstrates that the majority of lipid clusters do not predict differential patterns of grey matter degeneration as a function of CSF pTau/Aβ, highlighting the importance of unsaturated phosphatidylcholines and unsaturated acylcarnitines for grey matter integrity. The FDR-corrected salience map resulting from the unsaturated acylcarnitine whole-brain PLS analysis is shown in Fig. 6. Unsaturated acylcarnitines predict a spatial pattern of degeneration which consists of both overlapping and non-overlapping regions compared to the unsaturated phosphatidylcholines.

### Influence of age, BMI and sex on brain–lipid relationships

Prior work has shown that age, BMI and sex strongly influence the lipidome.^71,79,80^ Therfore, we assessed whether age, BMI or sex were driving the relationships observed between unsaturated phosphatidylcholines or unsaturated acylcarnitines and grey matter loss. We again conducted CSF group whole-brain grey matter PLS analyses, this time excluding age and BMI as input variables for each of these lipid clusters. When variance from age and BMI do not contribute to the multivariate relationship, unsaturated acylcarnitines do not significantly predict grey matter degeneration (*P* = 0.099 on 5000 permutations). In contrast, the unsaturated phosphatidylcholines alone predict differences in grey matter degeneration between the normal and abnormal CSF groups (*P* = 0.020 on 5000 permutations). The Sørensen–Dice coefficient was used to compare the similarity between the unsaturated phosphatidylcholine salience maps with and without age and BMI, where a value of 1 reflects two identical salience maps, and a value of 0 represents no overlap between the two salience maps. Because the Sørensen–Dice coefficient compares binary maps, we binarized each salience map at its FDR-corrected *z*-critical value prior to comparison. The Sørensen–Dice coefficient was 0.73, indicating that the expression of the phosphatidylcholine latent variable is highly similar with and without the inclusion of age and BMI. Thus, the observed relationship is largely driven by the unsaturated phosphatidylcholines, whereas the relationship between unsaturated acylcarnitines and grey matter degeneration is highly driven by interactions with age and BMI.

Finally, we investigated whether the relationship observed between unsaturated phosphatidylcholines and grey matter degeneration was driven by imbalances in the sexes within the normal and abnormal CSF groups (normal CSF % male = 45.2%, abnormal CSF % male = 61.5%, χ^2^ = 4.87, *P* = 0.034). To do so, we conducted PLS analyses where participants were grouped by biological sex, regardless of CSF pathology. In these analyses, a significant latent variable demonstrates that the relationship between grey matter degeneration and lipids in a cluster differs as a function of sex. Inputs to the sex PLS analysis were otherwise identical to the CSF analysis.

When grouping by sex, there was a significant latent variable that explained the differential relationship between unsaturated phosphatidylcholines and whole-brain grey matter degeneration in males and females (*P* = 0.0020 on 5000 permutations). The salience map (Supplementary Fig. 1) shows that there is sexual dimorphism in the relationship between unsaturated phosphatidylcholines and longitudinal grey matter integrity in regions including the temporal lobe, precuneus and frontal cortex but not the basal forebrain (Supplementary Fig. 1), suggesting that biological sex alone does not explain the full moderating effect of CSF pTau/Aβ pathology.

## Discussion

In agreement with several other studies in the field,^38,40,67,81–85^ we show a dysregulation of several lipid species in individuals with probable Alzheimer’s disease. Furthermore, we demonstrated a selective relationship between unsaturated phosphatidylcholines and degeneration of the basal forebrain which is modified by probable Alzheimer's disease, as indexed from CSF biomarkers of pTau and Aβ concentrations. We show that this relationship is not driven by age, BMI or sex. These results provide support for abnormal phosphatidylcholine metabolism as a contributing factor to the selective vulnerability of the basal forebrain in Alzheimer's disease. Though this idea was first proposed in the 1990s,^26^ a relationship between phosphatidylcholines and longitudinal basal forebrain degeneration has to our knowledge never been demonstrated in humans.

Our results underscore the importance of phosphatidylcholines to the grey matter integrity of the basal forebrain. Of the two phosphatidylcholine clusters identified by the ChemRICH approach (saturated and unsaturated), only the unsaturated phosphatidylcholines were dysregulated in the abnormal CSF group and predicted basal forebrain degeneration. Non-human animal studies suggest multiple roles of unsaturated phosphatidylcholines in normal and abnormal brain conditions. For instance, emerging evidence indicates that phosphatidylcholines containing polyunsaturated fatty acids can attenuate Aβ induced neurotoxicity in Alzheimer’s disease models by reducing inflammation and increasing autophagic clearance mechanisms.^86,87^ Additionally, dietary restriction of polyunsaturated fatty acids alters the composition of fatty acids on phosphatidylcholine in the brains of rodents. This is accompanied by decreased binding of cholinergic muscarinic receptors and altered acetylcholine release in the hippocampus.^88^ Arachidonic acid, a polyunsaturated fatty acid, facilitates acetylcholine release in cultured neurons regardless of whether it was endogenously produced or supplemented.^89^ Consistent with these results, we saw that the majority of the reliable phosphatidylcholines in our NbM PLS analyses contained polyunsaturated fatty acids. In humans, a large study of older adults examined the relationships of baseline serum metabolites with longitudinal ventricular expansion, cognitive decline, and conversion from mild cognitive impairment to Alzheimer’s disease.^85^ Of the six metabolites to show relationships with all of these outcome measures, three were unsaturated phosphatidylcholines. Moreover, serum unsaturated phosphatidylcholines were associated with pathological Aβ even in presymptomatic individuals, supporting an early role for phosphatidylcholine dysregulation in Alzheimer’s disease.

Our understanding of the mechanisms by which phosphatidylcholine levels become abnormal in Alzheimer’s disease is incomplete. However, two lines of evidence have pointed to links between Aβ pathology and phosphatidylcholine metabolic pathways. For instance, cell culture work indicates that accumulation of Aβ can decrease choline flux into the cell through its transporters for both acetylcholine synthesis^90^ and phospholipid synthesis.^91^ Importantly, Novakova *et al.*^91^ found that impairment of choline transport occurred at low (100 nm) concentrations of Aβ. Because free choline is a precursor for the synthesis of phosphatidylcholines^22,92^ and is additionally used to synthesize acetylcholine in cholinergic neurons,^93,94^ early accumulation of Aβ could create a bottleneck on resources necessary for both structural and functional maintenance of cholinergic neurons.

This observed relationship between Aβ and lipid dysregulation may be linked to APOE, the brain’s major lipid transporter. APOE is involved in both the clearance of Aβ^95,96^ and the transport of phosphatidylcholine,^97^ and carrying the ε4 allele of the *APOE* gene is the largest genetic risk factor for late-onset Alzheimer’s disease.^98,99^ Studies have also reported increased degeneration of the basal forebrain cholinergic system in *APOE4* carriers compared with non-carriers.^59,100^ Unsurprisingly, the abnormal CSF group contained the majority of the *APOE4* carriers in our sample, with only eight normal CSF individuals having one or more ε4 alleles. Because of the important role of APOE4 in the pathophysiology of Alzheimer’s disease and cholinergic dysfunction, it is possible that the combination of Alzheimer’s proteopathies and genetic risk conferred by *APOE4* combine to produce the strongest relationship between unsaturated phosphatidylcholines and basal forebrain degeneration. In line with this idea, the APOE4 protein induces lipid droplet formation in induced pluripotent stem cell-derived astrocytes and yeast, leading to morphological defects.^101^ These defects are specific to APOE4 (not resulting from Aβ) and are rescued by choline supplementation via increased phosphatidylcholine synthesis through the Kennedy pathway. Because of the importance of choline and phosphatidylcholine metabolism on APOE4-induced lipid dysfunction, we may therefore see that *APOE4* moderates the relationship between unsaturated phosphatidylcholines and basal forebrain degeneration in our abnormal CSF group. Although we did not have the sample size to examine interactions between *APOE4* and CSF, future large-scale studies could further investigate this relationship.

Examining the associations between single plasma analytes and pathological features of Alzheimer's disease has been vital in identifying lipid species that are involved in Alzheimer's disease. However, failing to account for the complex interrelationships that exist among lipids that are structurally or physiologically similar may cause lipid dysregulation which occurs at a network level to be overlooked. Previous studies in humans have linked various types of blood lipids to Alzheimer's disease clinical diagnosis, CSF proteopathies, cognitive scores and neuroimaging measures using univariate statistical methods.^67,68^ The present study extends upon previous human research by using multivariate PLS techniques and integrating multimodal data including serum, CSF, and longitudinal structural MRI within the same individuals. Our findings extend the work by Toledo *et al.*,^85^ who found differences in blood levels of phosphatidylcholines in individuals within the same cognitive group (e.g. cognitively normal, mild cognitive impairment or Alzheimer’s disease) when split by CSF Aβ. Together, these more recent findings stress the importance of integrating both CSF biomarkers and cognitive data to stratify patients when studying the human lipidome in Alzheimer’s disease.

### Limitations and future directions

A primary limitation of this study is the presence of both early and advanced stages of Alzheimer’s disease in our abnormal CSF group. Most participants with abnormal CSF in the MRI subset had a clinical diagnosis of mild cognitive impairment (*n* = 84) or Alzheimer’s disease (*n* = 52). Preclinical individuals (*n* = 25) represent a minority of this subset. We were therefore unable to stratify out preclinical individuals to investigate whether a relationship between unsaturated phosphatidylcholine levels and longitudinal basal forebrain degeneration exists at the earliest stage of the disease. Future studies can address this question with increased sample sizes and the incorporation of molecular imaging techniques. For example, PET imaging with the [^18^F]-FEOBV radiotracer can measure damage to cholinergic axons. Interestingly, axonal damage may precede damage to cholinergic cell bodies as quantified by structural MRI measurements of basal forebrain volume.^5,6^ [^18^F]-FEOBV binds selectively to the vesicular acetylcholine transporter,^102^ a protein that is expressed in cholinergic nerve terminals.^103,104^ [^18^F]-FEOBV PET in preclinical participants would therefore give an earlier, cell type-specific measurement of cholinergic damage which is lacking in the current study. Our longitudinal structural MRI measurements of basal forebrain volumetry measure degeneration in a region that is largely but not exclusively cholinergic.^55,105^ To mitigate this limitation, we included *a priori* region of interest analyses using the NbM which is composed of over 90% cholinergic neurons.^56^ Nonetheless, using [^18^F]-FEOBV PET to determine whether phosphatidylcholines predict cortical cholinergic denervation in cognitively normal older adults with pathological CSF pTau/Aβ warrants further research.

Finally, the conclusions that can be drawn about a relationship between unsaturated phosphatidylcholines and basal forebrain integrity would be strengthened by a lipidomics data set that was collected in the brain or CSF. Only serum lipidomics data was available in the ADNI Phase 1 population at the time of this study. Because of the separation of central and peripheral lipid metabolism,^106,107^ measurements of lipids in the serum may not be specifically related to the degeneration of vulnerable cell types in the brain, and instead more generally index the severity of Alzheimer’s disease. If this were the case, multiple serum lipid clusters would be expected to predict a common pattern of widespread neurodegeneration typical of Alzheimer’s disease. In our analyses, only one lipid cluster predicted longitudinal degeneration over and above age and BMI—unsaturated phosphatidylcholines—and this pattern was expressed in the basal forebrain nuclei and known targets of its projections. The biochemical and anatomical specificity of these findings argues against the possibility of blood–lipidome wide dysfunction predicting a general pattern of Alzheimer’s disease degeneration.

Overall, our study was to our knowledge the first to report that unsaturated phosphatidylcholines are related to longitudinal basal forebrain volumes in living adults with probable Alzheimer's disease. Our findings are in line with the hypothesis that cholinergic basal forebrain structural integrity is related to the availability of phosphatidylcholines, although the exact mechanism of this link in the central nervous system remains to be elucidated.

## Supplementary Material

fcac318_Supplementary_Data

## Abbreviations

Aβ =: amyloid beta
ADNI =: Alzheimer’s Disease Neuroimaging Initiative
APOE =: apolipoprotein E
BMI =: body mass index
chemRICH =: chemical similarity enrichment analysis
^18^F-FEOBV =: ^18^F-Fluoroethoxybenzovesamicol
FDR =: false discovery rate
KS =: Kolmogorov–Smirnov test
NbM =: nucleus basalis of Meynert
PLS =: partial least squares
pTau =: hyperphosphorylated tau
