# Supplementary material for: Serum unsaturated phosphatidylcholines predict longitudinal basal forebrain degeneration in Alzheimer’s disease
Source: Brain Commun. 2022 Dec 2;4(6):fcac318. doi: 10.1093/braincomms/fcac318 (PMC10103184; doi:10.1093/braincomms/fcac318)
Supplement: fcac318_Supplementary_Data [file fcac318_supplementary_data.pdf]

Supplementary Table 1 Inputs to nucleus basalis of Meynert partial least squares analysis

Unique ID	Variable name	Group	Latent variable correlation	Bootstrapped confidence interval
UCD.Lipid.163	PC p-40:6	abnormal CSF	0.2367	[-0.0061, 0.4504]
UCD.Lipid.389	PC(16:0_18:2)	abnormal CSF	0.2187	[0.0464, 0.3806]
UCD.Lipid.150	PC 39:6	abnormal CSF	0.2076	[0.0025, 0.3825]
UCD.Lipid.392	PC(17:0_18:1)	abnormal CSF	0.2030	[0.0150, 0.3886]
UCD.Lipid.426	PC(P-22:0_20:4)	abnormal CSF	0.1995	[0.0005, 0.3894]
UCD.Lipid.408	PC(18:0_22:5)	abnormal CSF	0.1864	[-0.0007, 0.3518]
UCD.Lipid.422	PC(P-18:0_20:4)	abnormal CSF	0.1853	[-0.0497, 0.3970]
UCD.Lipid.423	PC(P-18:0_20:5)	abnormal CSF	0.1709	[-0.0355, 0.3596]
UCD.Lipid.393	PC(17:0_18:2)	abnormal CSF	0.1634	[0.0597, 0.2616]
UCD.Lipid.385	PC(15:0_18:1)	abnormal CSF	0.1591	[0.0458, 0.2857]
UCD.Lipid.428	PC p-44:4	abnormal CSF	-0.1564	[-0.2564, -0.0289]
UCD.Lipid.146	PC 38:4 B	abnormal CSF	0.1530	[0.0511, 0.2619]
UCD.Lipid.406	PC 38:6	abnormal CSF	0.1529	[0.0526, 0.2725]
UCD.Lipid.165	PC p-42:3	abnormal CSF	0.1475	[0.0160, 0.2651]
UCD.Lipid.151	PC(18:0_22:5)	abnormal CSF	0.1470	[0.0440, 0.2440]
UCD.Lipid.407	PC(18:0_22:4)	abnormal CSF	0.1444	[0.0335, 0.2497]
UCD.Lipid.141	PC(18:1_18:2)	abnormal CSF	0.1417	[0.0498, 0.2504]
UCD.Lipid.152	PC(18:0_22:6)	abnormal CSF	0.1411	[0.0593, 0.2271]
UCD.Lipid.390	PC(16:0_18:3)	abnormal CSF	0.1410	[0.0252, 0.2547]
UCD.Lipid.409	PC(18:0_22:6)	abnormal CSF	0.1408	[-0.0028, 0.2776]
UCD.Lipid.396	PC(18:0_18:2)	abnormal CSF	0.1406	[-0.0350, 0.3156]
UCD.Lipid.143	PC 36:6	abnormal CSF	0.1373	[0.0662, 0.2269]
UCD.Lipid.383	PC(16:0_16:1)	abnormal CSF	0.1339	[0.0259, 0.2536]
UCD.Lipid.404	PC(18:0_20:4)	abnormal CSF	0.1328	[0.0379, 0.2349]
UCD.Lipid.403	PC(18:0_20:3)	abnormal CSF	0.1274	[0.0434, 0.2253]
UCD.Lipid.425	PC(P-20:0_20:4)	abnormal CSF	0.1242	[-0.1158, 0.3352]
UCD.Lipid.395	PC(18:0_18:1)	abnormal CSF	0.1233	[-0.0495, 0.2655]
UCD.Lipid.384	PC(14:0_18:2)	abnormal CSF	0.1228	[-0.0016, 0.2289]
UCD.Lipid.145	PC 37:6	abnormal CSF	0.1182	[0.0118, 0.2310]
UCD.Lipid.400	PC(19:0_18:2)	abnormal CSF	0.1175	[-0.0543, 0.2714]
UCD.Lipid.149	PC 38:7	abnormal CSF	0.1172	[0.0203, 0.2348]
UCD.Lipid.147	PC(16:0_22:5)	abnormal CSF	0.1146	[0.0375, 0.2202]
UCD.Lipid.164	PC p-40:7	abnormal CSF	0.1063	[0.0073, 0.2324]
UCD.Lipid.402	PC(20:0_18:2)	abnormal CSF	0.1028	[-0.0776, 0.2738]
UCD.Lipid.137	PC 31:1	abnormal CSF	0.0934	[-0.0432, 0.2117]
UCD.Lipid.405	PC(18:1_20:4)	abnormal CSF	0.0931	[-0.0164, 0.1909]
UCD.Lipid.397	PC(18:1_18:2)	abnormal CSF	0.0926	[-0.0148, 0.2336]
UCD.Lipid.160	PC p-40:1	abnormal CSF	0.0899	[-0.0193, 0.2018]
UCD.Lipid.399	PC(16:1_20:4)	abnormal CSF	0.0896	[-0.1041, 0.2779]
UCD.Lipid.386	PC(16:0_18:2)	abnormal CSF	0.0892	[-0.0376, 0.2094]
UCD.Lipid.394	PC(15:0_20:4)	abnormal CSF	0.0875	[-0.0620, 0.2082]

UCD.Lipid.148	PC(16:0_22:6	abnormal CSF	0.0851	[-0.0068, 0.1794]
UCD.Lipid.418	PC(P-18:0_18:2)	abnormal CSF	-0.0820	[-0.1643, 0.0135]
UCD.Lipid.401	PC(17:0_20:4)	abnormal CSF	0.0813	[-0.0458, 0.1988]
UCD.Lipid.135	PC 30:1	abnormal CSF	0.0808	[-0.0824, 0.2447]
UCD.Lipid.159	PC(P-18:0_20:4)	abnormal CSF	0.0800	[-0.0283, 0.1963]
UCD.Lipid.419	PC(P-16:0_20:3)	abnormal CSF	0.0785	[-0.1047, 0.2366]
UCD.Lipid.398	PC(16:0_20:4)	abnormal CSF	0.0783	[-0.0462, 0.1856]
UCD.Lipid.158	PC p-38:2	abnormal CSF	0.0757	[-0.0518, 0.1976]
AGE	AGE	abnormal CSF	0.0695	[-0.0333, 0.1797]
UCD.Lipid.414	PC(P-18:0_16:0)	abnormal CSF	-0.0694	[-0.1666, 0.0261]
UCD.Lipid.154	PC 42:6	abnormal CSF	0.0665	[-0.0463, 0.1789]
UCD.Lipid.162	PC p-40:6	abnormal CSF	0.0623	[-0.0507, 0.1760]
UCD.Lipid.388	PC(16:0_18:1)	abnormal CSF	0.0583	[-0.0561, 0.1921]
UCD.Lipid.156	PC p-32:1	abnormal CSF	0.0514	[-0.0794, 0.1729]
UCD.Lipid.153	PC 42:5	abnormal CSF	0.0462	[-0.0402, 0.1471]
BMI	BMI	abnormal CSF	0.0420	[-0.0981, 0.1871]
UCD.Lipid.391	PC(14:0_20:4)	abnormal CSF	0.0368	[-0.0815, 0.1369]
UCD.Lipid.416	PC(P-16:0_18:2)	abnormal CSF	-0.0340	[-0.1566, 0.0717]
UCD.Lipid.142	PC(18:2_18:2)	abnormal CSF	0.0332	[-0.0725, 0.1440]
UCD.Lipid.410	PC(18:1_22:6)	abnormal CSF	-0.0302	[-0.1563, 0.0794]
UCD.Lipid.157	PC(P-16:0_18:1)	abnormal CSF	-0.0292	[-0.1440, 0.0857]
UCD.Lipid.417	PC(P-18:0_18:1)	abnormal CSF	0.0276	[-0.0728, 0.1361]
UCD.Lipid.415	PC(P-16:0_18:1)	abnormal CSF	0.0246	[-0.1457, 0.1572]
UCD.Lipid.138	PC 32:3	abnormal CSF	0.0180	[-0.0963, 0.1245]
UCD.Lipid.411	PC(18:2_22:6)	abnormal CSF	-0.0116	[-0.1289, 0.0837]
UCD.Lipid.470	SM(d18:1_18:0)	abnormal CSF	0.0082	[-0.0901, 0.1134]
UCD.Lipid.144	PC 37:3	abnormal CSF	0.0073	[-0.0871, 0.1143]
UCD.Lipid.161	PC p-40:5	abnormal CSF	0.0065	[-0.0965, 0.1146]
UCD.Lipid.421	PC(P-18:0_20:3)	abnormal CSF	0.0044	[-0.1399, 0.1380]
UCD.Lipid.420	PC(P-16:0_20:4)	abnormal CSF	0.0037	[-0.1588, 0.1489]
UCD.Lipid.427	PC p-42:5	abnormal CSF	-0.0026	[-0.0925, 0.1027]
UCD.Lipid.163	PC p-40:6	normal CSF	-0.0436	[-0.3407, 0.2049]
UCD.Lipid.389	PC(16:0_18:2)	normal CSF	-0.0839	[-0.3702, 0.1769]
UCD.Lipid.150	PC 39:6	normal CSF	-0.0506	[-0.2799, 0.1296]
UCD.Lipid.392	PC(17:0_18:1)	normal CSF	-0.0906	[-0.4160, 0.1777]
UCD.Lipid.426	PC(P-22:0_20:4)	normal CSF	-0.0663	[-0.3632, 0.1949]
UCD.Lipid.408	PC(18:0_22:5)	normal CSF	-0.0293	[-0.2548, 0.1569]
UCD.Lipid.422	PC(P-18:0_20:4)	normal CSF	-0.0081	[-0.3450, 0.2793]
UCD.Lipid.423	PC(P-18:0_20:5)	normal CSF	-0.0439	[-0.3562, 0.2223]
UCD.Lipid.393	PC(17:0_18:2)	normal CSF	-0.0909	[-0.3715, 0.1660]
UCD.Lipid.385	PC(15:0_18:1)	normal CSF	-0.1747	[-0.4208, 0.0723]
UCD.Lipid.428	PC p-44:4	normal CSF	0.1949	[0.0018, 0.3832]
UCD.Lipid.146	PC 38:4 B	normal CSF	-0.2058	[-0.5015, 0.0776]
UCD.Lipid.406	PC(18:1_20:5)_PC(18:2	normal CSF	-0.1231	[-0.3522, 0.0715]
UCD.Lipid.165	PC p-42:3	normal CSF	0.0245	[-0.2415, 0.2416]

UCD.Lipid.151	PC(18:0_22:5)	normal CSF	0.0767	[-0.2111, 0.3356]
UCD.Lipid.407	PC(18:0_22:4)	normal CSF	-0.1393	[-0.3832, 0.0754]
UCD.Lipid.141	PC(18:1_18:2)	normal CSF	-0.0495	[-0.2968, 0.1389]
UCD.Lipid.152	PC(18:0_22:6)	normal CSF	-0.0257	[-0.2785, 0.1527]
UCD.Lipid.390	PC(16:0_18:3)	normal CSF	-0.2103	[-0.4114, -0.0288]
UCD.Lipid.409	PC(18:0_22:6)	normal CSF	-0.1128	[-0.4412, 0.1296]
UCD.Lipid.396	PC(18:0_18:2)	normal CSF	-0.1880	[-0.4882, 0.0561]
UCD.Lipid.143	PC 36:6	normal CSF	-0.0636	[-0.3039, 0.1220]
UCD.Lipid.383	PC(16:0_16:1)	normal CSF	-0.2020	[-0.3884, -0.0117]
UCD.Lipid.404	PC(18:0_20:4)	normal CSF	-0.2184	[-0.4438, -0.0253]
UCD.Lipid.403	PC(18:0_20:3)	normal CSF	-0.1344	[-0.3768, 0.0349]
UCD.Lipid.425	PC(P-20:0_20:4)	normal CSF	-0.0192	[-0.3004, 0.2177]
UCD.Lipid.395	PC(18:0_18:1)	normal CSF	0.1149	[-0.1750, 0.3550]
UCD.Lipid.384	PC(14:0_18:2)	normal CSF	0.0388	[-0.2503, 0.3041]
UCD.Lipid.145	PC 37:6	normal CSF	-0.1193	[-0.4195, 0.1187]
UCD.Lipid.400	PC(19:0_18:2)	normal CSF	0.0515	[-0.2385, 0.3084]
UCD.Lipid.149	PC 38:7	normal CSF	-0.0940	[-0.3829, 0.1401]
UCD.Lipid.147	PC(16:0_22:5)	normal CSF	-0.0371	[-0.2369, 0.0937]
UCD.Lipid.164	PC p-40:7	normal CSF	-0.0819	[-0.2942, 0.1277]
UCD.Lipid.402	PC(20:0_18:2)	normal CSF	-0.0829	[-0.3892, 0.1430]
UCD.Lipid.137	PC 31:1	normal CSF	0.0991	[-0.2440, 0.3840]
UCD.Lipid.405	PC(18:1_20:4)	normal CSF	-0.0686	[-0.3347, 0.1610]
UCD.Lipid.397	PC(18:1_18:2)	normal CSF	-0.1985	[-0.3893, -0.0325]
UCD.Lipid.160	PC p-40:1	normal CSF	-0.1144	[-0.3758, 0.0918]
UCD.Lipid.399	PC(16:1_20:4)	normal CSF	-0.1345	[-0.3791, 0.0806]
UCD.Lipid.386	PC(16:0_18:2)	normal CSF	-0.0143	[-0.3850, 0.3250]
UCD.Lipid.394	PC(15:0_20:4)	normal CSF	0.0575	[-0.3489, 0.4108]
UCD.Lipid.148	PC(16:0_22:6	normal CSF	-0.0750	[-0.3187, 0.1168]
UCD.Lipid.418	PC(P-18:0_18:2)	normal CSF	-0.2128	[-0.4882, -0.0102]
UCD.Lipid.401	PC(17:0_20:4)	normal CSF	0.0773	[-0.1776, 0.3014]
UCD.Lipid.135	PC 30:1	normal CSF	-0.0206	[-0.2947, 0.2291]
UCD.Lipid.159	PC(P-18:0_20:4)	normal CSF	-0.0912	[-0.3911, 0.1473]
UCD.Lipid.419	PC(P-16:0_20:3)	normal CSF	-0.0514	[-0.3877, 0.2286]
UCD.Lipid.398	PC(16:0_20:4)	normal CSF	0.0773	[-0.2242, 0.3443]
UCD.Lipid.158	PC p-38:2	normal CSF	0.0355	[-0.2496, 0.2789]
AGE	AGE	normal CSF	-0.0743	[-0.3260, 0.1502]
UCD.Lipid.414	PC(P-18:0_16:0)	normal CSF	-0.0245	[-0.3096, 0.2248]
UCD.Lipid.154	PC 42:6	normal CSF	-0.0306	[-0.2887, 0.1921]
UCD.Lipid.162	PC p-40:6	normal CSF	-0.0801	[-0.3529, 0.1374]
UCD.Lipid.388	PC(16:0_18:1)	normal CSF	-0.0046	[-0.2822, 0.2583]
UCD.Lipid.156	PC p-32:1	normal CSF	-0.0140	[-0.3324, 0.2250]
UCD.Lipid.153	PC 42:5	normal CSF	0.1555	[-0.1477, 0.3833]
BMI	BMI	normal CSF	0.0841	[-0.1332, 0.2572]
UCD.Lipid.391	PC(14:0_20:4)	normal CSF	0.0632	[-0.2397, 0.3283]
UCD.Lipid.416	PC(P-16:0_18:2)	normal CSF	-0.1292	[-0.4416, 0.1483]

UCD.Lipid.142	PC(18:2_18:2)	normal CSF	0.1015	[-0.1887, 0.3559]
UCD.Lipid.410	PC(18:1_22:6)	normal CSF	-0.0411	[-0.3491, 0.2527]
UCD.Lipid.157	PC(P-16:0_18:1)	normal CSF	-0.1360	[-0.4413, 0.0961]
UCD.Lipid.417	PC(P-18:0_18:1)	normal CSF	-0.2292	[-0.5193, 0.0299]
UCD.Lipid.415	PC(P-16:0_18:1)	normal CSF	0.0797	[-0.2499, 0.3779]
UCD.Lipid.138	PC 32:3	normal CSF	0.0240	[-0.2864, 0.2925]
UCD.Lipid.411	PC(18:2_22:6)	normal CSF	0.0122	[-0.2673, 0.2561]
UCD.Lipid.470	SM(d18:1_18:0)	normal CSF	-0.1457	[-0.3310, 0.0471]
UCD.Lipid.144	PC 37:3	normal CSF	-0.0110	[-0.2535, 0.1760]
UCD.Lipid.161	PC p-40:5	normal CSF	-0.1325	[-0.4041, 0.0859]
UCD.Lipid.421	PC(P-18:0_20:3)	normal CSF	-0.1493	[-0.4606, 0.0878]
UCD.Lipid.420	PC(P-16:0_20:4)	normal CSF	-0.1419	[-0.4882, 0.1670]
UCD.Lipid.427	PC p-42:5	normal CSF	0.1300	[-0.1537, 0.3549]

95% bootstrapped confidence intervals are indicated as [lower bound, upper bound]. Lipids are named according to their headgroup (phosphatidylcholine (PC)) and total carbons and double bonds (carbons:double bonds) on their acyl chains. Acyl chain information is indicated for lipids when available. BMI = body mass index

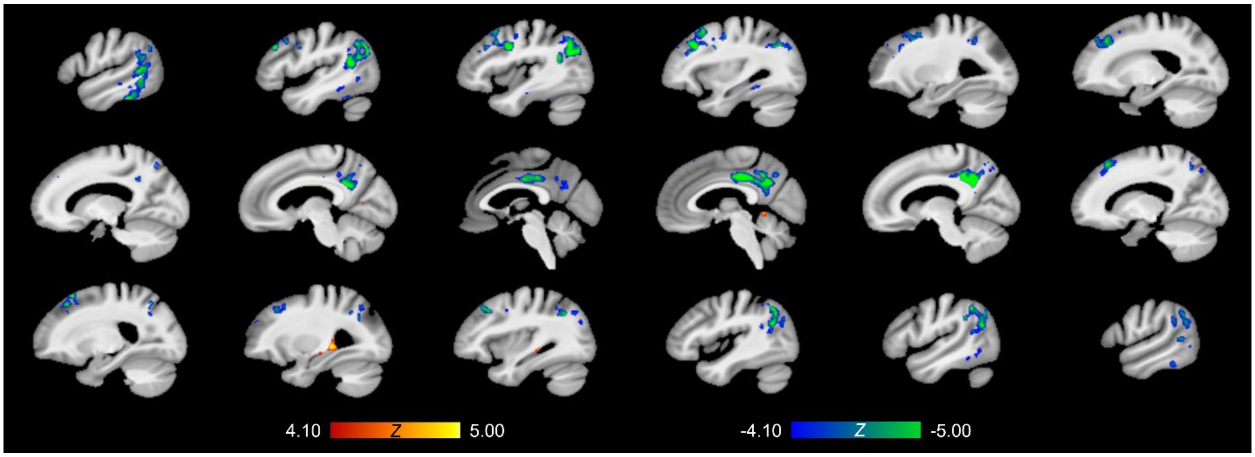

Supplementary Figure 1 Unsaturated phosphatidylcholines predict sex differences in grey matter degeneration. Unsaturated phosphatidylcholines predict sex differences in longitudinal grey matter degeneration in voxels shown in warm or cool tones (latent variable p = 0.0020) through a whole brain grey matter PLS analysis. Expression of this latent variable is absent in the basal forebrain. Only voxels with a $p < 0.05$ after False Discovery Rate correction for all voxels in the grey matter are shown.
